# LYSOSOMAL SIGNALS IN LONGEVITY REGULATION ACROSS THE SCALE

**DOI:** 10.1093/geroni/igad104.1133

**Published:** 2023-12-21

**Authors:** Meng Wang

**Affiliations:** Janelia Research Campus, Ashburn, Virginia, United States

## Abstract

Lysosomes are key organelles in the cell that constitute an acidic subcellular environment and contain approximately 60 different types of hydrolytic enzymes. With the aid of these acidic hydrolytic enzymes, lysosomes are highly metabolically active and can digest various macromolecules delivered through endocytosis, phagocytosis, and autophagy. Moreover, lysosomes function as a “signaling hub” that integrates metabolic inputs, organelle interaction, and longevity control. Our studies discovered a pro-longevity lysosomal acidic lipase that activates a lysosome-to-nucleus retrograde lipid signaling pathway in Caenorhabditis elegans, and in turn revealed the critical role of this lysosomal lipid signaling in promoting oxidative stress tolerance and lipid catabolism through tuning mitochondrial activities. Furthermore, we discovered that this lysosomal lipase induces the release of lipid messengers from peripheral metabolic tissues, which act on neurons and lead to the up-regulation of the neuropeptide signaling pathway by activating a nuclear hormone receptor. More recently, lysosome-specific proteomic profiling has revealed that AMPK is specifically recruited to the lysosome upon lysosomal lipolysis and mediates the longevity effect, suggesting a previously unknown interaction between AMPK and lysosomal lipid signaling. Together, these findings highlight the crucial role of lysosomal signals in actively coordinating organelle crosstalk and tissue communication to improve longevity and promote healthy aging.

